# A cost-consequence analysis of a nudge intervention to improve hospital care of older people at the end of life: results from a stepped-wedge cluster randomised trial

**DOI:** 10.1093/ageing/afaf280

**Published:** 2025-10-06

**Authors:** Hannah E Carter, Thomasina Donovan, Nicole M White, Xing J Lee, Christine Brown, Nicholas Graves, Steven McPhail, Magnolia Cardona, Ben P White, Lindy Willmott, Gillian Harvey, Leonie Callaway, Ken Hillman, Adrian G Barnett

**Affiliations:** Australian Centre for Health Services Innovation and Centre for Healthcare Transformation, School of Public Health and Social Work, Faculty of Health, Queensland University of Technology, Kelvin Grove, Queensland, Australia; Australian Centre for Health Services Innovation and Centre for Healthcare Transformation, School of Public Health and Social Work, Faculty of Health, Queensland University of Technology, Kelvin Grove, Queensland, Australia; Australian Centre for Health Services Innovation and Centre for Healthcare Transformation, School of Public Health and Social Work, Faculty of Health, Queensland University of Technology, Kelvin Grove, Queensland, Australia; Australian Centre for Health Services Innovation and Centre for Healthcare Transformation, School of Public Health and Social Work, Faculty of Health, Queensland University of Technology, Kelvin Grove, Queensland, Australia; Australian Centre for Health Services Innovation and Centre for Healthcare Transformation, School of Public Health and Social Work, Faculty of Health, Queensland University of Technology, Kelvin Grove, Queensland, Australia; Duke-NUS Postgraduate Medical School, National University of Singapore, 8 College Rd, Singapore 169857, Singapore; Australian Centre for Health Services Innovation and Centre for Healthcare Transformation, School of Public Health and Social Work, Faculty of Health, Queensland University of Technology, Kelvin Grove, Queensland, Australia; University of New South Wales, School of Population Health, Kensington, New South Wales, Australia; Bond University, Institute for Evidence-Based Healthcare, Robina, Queensland, Australia; Faculty of Business and Law, Australian Centre for Health Law Research, School of Law, Queensland University of Technology, Brisbane, Queensland, Australia; Faculty of Business and Law, Australian Centre for Health Law Research, School of Law, Queensland University of Technology, Brisbane, Queensland, Australia; Australian Centre for Health Services Innovation and Centre for Healthcare Transformation, School of Public Health and Social Work, Faculty of Health, Queensland University of Technology, Kelvin Grove, Queensland, Australia; Flinders University, College of Nursing and Health Sciences, Bedford Park, South Australia, Australia; Royal Brisbane and Women’s Hospital, Herston, Queensland, Australia; Faculty of Health, Queensland University of Technology, Kelvin Grove, Queensland, Australia; Faculty of Medicine, The University of Queensland, Herston, Queensland, Australia; The Simpson Centre for Health Services Research, SWS Clinical School, Ingham Institute for Applied Medical Research, Sydney, New South Wales, Australia; Intensive Care Unit, Liverpool Hospital, Sydney, New South Wales, Australia; Australian Centre for Health Services Innovation and Centre for Healthcare Transformation, School of Public Health and Social Work, Faculty of Health, Queensland University of Technology, Kelvin Grove, Queensland, Australia

**Keywords:** costs, interventions, hospitals, at risk, InterACT, older people

## Abstract

**Objectives:**

The ‘Intervention for Appropriate Care and Treatment’ (InterACT) was a nudge intervention to identify hospital patients at risk of imminent death or deterioration and communicate this information to treating clinical teams. The aim was to improve the quality of care delivered. This paper reports a cost-consequence analysis of the InterACT intervention.

**Methods:**

A stepped-wedge cluster randomised trial was conducted across three large tertiary hospitals in Australia between May 2020 and June 2021. The cost of implementing the intervention was determined using prospectively collected staff time sheets, study documentation and field notes. Changes to hospital admission costs and health service outcomes between the trial’s intervention and control phases are also reported. Hospital admissions costs and other health service outcomes were obtained from hospital databases and patient chart reviews.

**Results:**

The mean intervention cost was $A 72 per at-risk patient admission identified. Additional site-level implementation costs ranged between $21 373 to $34 867 per hospital site, translating to $23 per at-risk admission. The intervention did not reduce the cost of intensive care unit admission, length of stay, medical emergency calls or in-hospital deaths. Wide confidence intervals around at-risk admission cost differences (95% CI: –$2264 to $3312) indicated there was large uncertainty.

**Conclusions:**

This cost-consequence analysis found that the intervention was not effective in reducing the cost of non-beneficial treatment, which is consistent with the broader InterACT results. This simple nudge-intervention alone may not be sufficient to impact health service resource use and costs in the complex end-of-life setting.

## Key Points

- While InterACT was a relatively low-cost intervention, it did not impact on overall hospital admission costs.
- A key strength of the analysis is the detailed costing of implementation strategies and activities.
- The study is limited by the use health service outcomes as proxy measures of care quality.
- Future studies should prioritise use of patient reported outcomes and consider the consumer voice.

## Introduction

Health care interventions at the end of life can be unnecessary [1–3] and may even increase suffering [4, 5]. Up to one in three people who die in hospital have been found to receive inappropriate treatments that confer no meaningful benefit [1]. While classifying treatments as ‘non-beneficial’ is often subjective and highly context-specific, some examples include ordering tests which will not affect treatment decisions, medications that are unlikely to alter patient outcomes, as well as use of active measures such as dialysis, radiotherapy, transfusions in the last few months of terminally ill patients’ lives [1]. Other examples include resuscitations and life support treatments which will not result in improved survival or quality of life or are inconsistent with patient or family preferences [1, 6]. These non-beneficial treatments are distressing to patients, family members and medical staff [7], and are a misuse of limited healthcare resources [8].

The Intervention for Appropriate Care and Treatment (InterACT) trial was designed to improve appropriate care and treatment decisions for older people admitted to hospital at the end of life [9]. The intervention was designed as a ‘nudge’ [10] that flagged at-risk patients and communicated their risk profiles to treating clinical teams, to prompt behaviour change. Results of the trial on admission outcomes [11] and care review outcomes [12] have been published separately, with most outcomes showing no effect.

The aim of this paper is to report a cost-consequence analysis of the InterACT intervention. The cost-consequence analysis framework provides an approach to transparently report and synthesise the full scope of intervention costs and consequences, allowing decision makers to form their own assessment of an intervention’s relative value for money [13].

## Methods

### The InterACT trial

This economic evaluation was conducted alongside a stepped-wedge cluster randomised trial in south-east Queensland, Australia. The trial was pre-registered (ACTRN12619000675123) and the study design and analyses were pre-specified in a published protocol [9].

The InterACT trial was conducted in three tertiary metropolitan public hospitals between May 2020 and June 2021. The trial design is illustrated in Supplementary Fig. S1. Each hospital represented a cluster; clusters were randomised to sequential timing of intervention commencement. All hospitals commenced the trial in the ‘control’ condition, which was defined as usual care. This was followed by a four-week implementation establishment phase, during which the changes to practice were discussed, and elements of the intervention were tailored to meet the needs of clinical teams. The ‘intervention’ condition commenced for each hospital immediately after the end of the implementation establishment phase. Hospitals then remained in the intervention phase until the end of the trial, between 16 and 35 weeks.

Throughout the trial, independent clinical auditors prospectively screened all patients aged 75 years and older admitted under the 14 enrolled clinical teams. The screening process occurred twice weekly and involved the completion of two validated tools, Criteria for Screening and Triaging to Appropriate aLternative care (CriSTAL) [14] and Supportive & Palliative Care Indicators Tool [15], designed to identify short-term risks of death and deterioration, respectively. Additional information on the screening process is reported in the Supplementary File S1. During the intervention phase, clinical teams were notified of any at-risk patients under their care. Notifications were provided through a tailored feedback mechanism that involved two flags: a real-time notification on the patient’s record and an email sent to clinicians about the at-risk patients at the end of each screening day. The intervention was designed as a ‘nudge’ and did not provide further guidance or instruction regarding the care provided to at-risk patients.

### Ethical considerations

Ethics approval was obtained from the Royal Brisbane and Women’s Hospital Human Research Ethics Committee (HREC) (Approval HREC/2019/QRBW/51606) and the QUT HREC (Approval 1,900,000,630). These approvals include a waiver of consent for access to de-identified patient and health service data. A Public Health Act (PHA) approval was obtained for access to patient data linkage (RD008146).

### Data collection

Costs associated with the InterACT intervention were in two categories: those associated with core intervention delivery (intervention costs) as well those reflecting ancillary activities that helped to support the intervention’s establishment, and ongoing implementation (implementation costs). This classification was intended to capture the real-world complexities of implementing new processes in complex hospital environments, which often requires time and resources beyond those needed to deliver the core intervention components. Our approach was grounded in theories from implementation science, which is concerned with the methods to promote the systematic uptake of evidence-based practice into routine practice [16].

All costs were estimated from the hospital perspective and are reported in 2024 Australian dollars. This reflects the nature of the Australian health care system where hospital admissions are publicly funded. Discounting was not required as all costs occurred within the relatively short 14-month time horizon of the trial.

#### Intervention costs

Intervention costs accounted for the time clinical nurse auditors took to complete the admission screening process. Auditor time was prospectively recorded in staff time sheets and costed using project invoices. The initial at-risk notification flags were activated by the clinical auditors at the time of screening, using a simple on-line trigger. The second notification was an automated email sent at the end of each screening day to the senior clinician who the patient was admitted under. Neither notification required additional resourcing beyond the initial software programming (included as implementation costs) and nurse auditor time to enter the data. The time taken for clinical teams to discuss and action any notifications was not included in this analysis, as it was assumed these activities fell within the existing scope of practice for these roles.

#### Implementation costs

Throughout the trial, additional resources were required to support the implementation of the intervention. A project manager helped to facilitate implementation with key activities including: meetings with senior hospital decision makers to secure executive buy-in for the intervention; establishing an Executive Advisory Group at each hospital to provide guidance and oversight of the intervention; conducting context assessments; providing training and support to clinical auditors, including conducting inter-rater reliability testing to ensure consistency in the screening process; communicating with clinical teams and other key stakeholders; and addressing general issues as they arose. Multiple hospital staff members were also involved in supporting activities including attending meetings, database programming and trouble-shooting, and assisting with auditor training and monitoring activities. Physical materials and travel to the sites to execute these activities were also required.

Implementation costs were collated using the Costing Implementation Strategies (Cost-IS) instrument [17]. This tool applies activity-based micro costing methods that allows for the synthesis of costs across relevant implementation categories. Data were sourced from study documents including study protocol, project timeline, completed context assessment documents, meeting minutes, demographic data from interviews and participant surveys and detailed field notes that were recorded by the project manager. Labour costs were valued from publicly available salary rates, while non-labour costs were valued using project invoices.

#### Hospital admissions costs

Hospital admission costs were included in the analysis to reflect the potential resource use impacts of the intervention on care delivery. Costs were included for all at-risk admissions as well as any subsequent readmissions that occurred within 12 weeks. This reflected the pre-specified trial protocol, which included 12-week readmissions as a secondary outcome. Patient-level admission costs were retrospectively extracted from hospital databases.

#### Outcomes

Outcomes selected for the cost-consequence analysis were consistent with InterACT trial outcomes and reflected proxy indicators of non-beneficial treatment. The primary outcome of the trial was intensive care unit (ICU) admission, which was selected due to the potential for these admissions to coincide with highly invasive treatments that unnecessarily increase suffering at the end of life [11]. Secondary outcomes were death in hospital, medical emergency call [18], readmissions within 12 weeks of discharge, clinician-led care review discussions, review of care directive measures and specialist palliative care referral. Data to inform these outcomes were obtained from routinely collected hospital records and patient chart audits for all at-risk patients identified in the trial.

### Analysis methods

Data management and analyses were conducted using R version 4.1.0 and MS Excel. The main R packages used for analyses were ‘tidyverse’, ‘lme4’ and ‘boot’. The analysis of intervention costs and outcomes was limited to patients at-risk and excluded an eight week trial suspension period that was required due to impacts of COVID-19. All results are presented using blinded hospital names.

Intervention and implementation costs were analysed descriptively at the hospital level. Implementation activity costs were additionally mapped to broad implementation strategies using the Expert Recommendations for Implementing Change framework [19].

Analyses of hospital admissions cost differences were conducted in adherence with established recommendations for economic evaluations conducted alongside stepped-wedge cluster randomised trials [20–22]. A generalised linear mixed model with Gamma distribution was applied because of the positive skew in costs. The model adjusted for trial phase (intervention or control) and calendar time as fixed effects, and cluster (hospital site) as a random effect. Calendar time was defined as the number of weeks since the start of the trial, based on patients’ date of hospital admission. The model also adjusted for patient age and gender as fixed effects, consistent with the trial’s main outcome analyses. Results are reported as unadjusted and adjusted mean differences (intervention minus control) with 95% confidence intervals (95% CI) obtained from parametric bootstrapping.

Detailed summaries of the statistical methods used for admission and care review outcomes have been reported separately [11, 12].

## Results

### At-risk admissions

A total of 7293 admissions were screened over the duration of the trial, of which 4268 were classified as ‘at-risk’ and eligible for inclusion in the final analysis. The median age of admitted patients was 84 years, and 52% were female. Admissions were evenly distributed between the control (50.2%) and intervention (48.8%) phases. The median length of stay was 5.8 days (Q1 to Q3: 3.2 to 11.1) in the control phase and 6.0 (Q1 to Q3: 3.3 to 12.4) in the intervention phase. Further details on the characteristics of at-risk admissions are provided in Supplementary Table S1.

### Intervention costs

Table 1 outlines the resource use and costs associated with delivering the InterACT intervention. Throughout the intervention phase, auditors screened an average of 136 admissions per week across the three study hospitals. Of these, 58% were classified as at-risk. The at-risk rate varied from 41% at Hospital Y to 66% at Hospital X.

**Table 1:** Intervention resource use and costs by individual hospital and combined

Mean resource use and cost components	Hospital X	Hospital Y	Hospital Z	All hospitals combined
Admissions screened per week	54	27	54	136
At-risk admissions identified per week (%)	35 (66%)	11 (41%)	33 (62%)	79 (58%)
Weekly auditor hours	35	18	34	86
Weekly auditor cost (AUD)	2291	1170	2244	5705
**Screened admission outcomes**				
Auditor hours per screened admission	0.65	0.67	0.63	0.63
Auditor costs per screened admission (AUD)	42	43	42	42
**At-risk admission outcomes**				
Auditor hours per at-risk admission	0.99	1.6	1.02	1.09
Auditor costs per at-risk admission (AUD)	65	106	68	72

Auditors spent a mean 0.63 hours per admission screened, which translated to 1.09 hours per at-risk admission. Costs followed a similar trend, with a mean cost of $42 per admission screened, and $72 when estimated per at-risk admission. While mean costs per admission screened remained relatively consistent across the three study hospitals, the mean costs per at-risk admission ranged from $65 in Hospital X to $106 in Hospital Y. This variation reflected differences in the underlying proportion of at-risk admissions between hospitals, with a relatively higher volume of records needing to be screened for each at-risk admission identified at Hospital Y.

### Implementation costs

Table 2 reports the total implementation costs by strategy across the three hospitals, including the relative cost per at-risk admission over the intervention period. All cost categories comprised activities that occurred at more than one time point throughout the implementation. Facilitation activities accounted for 52% of total implementation costs across the three hospitals, which included activities related to auditor training, monitoring, communication and inter-rater reliability testing. Local Executive Advisory Group meetings accounted for a further 27% of implementation costs, followed by strategies to adapt and tailor the intervention to local contexts (19%) and support for local site champions (2%). The total implementation cost per at risk admission was $47, with variation observed across the three hospitals reflecting the relatively smaller number of at-risk admissions at Hospital Y.

**Table 2:** Implementation costs by strategy

Implementation cost categories	Hospital X		Hospital Y		Hospital Z		All hospitals combined	
	Total	Per at-risk admission	Total	Per at-risk admission	Total	Per at-risk admission	Total	Per at-risk admission
**Adapt and tailor to context**	**$8455**	**$8.97**	**$5190**	**$17.37**	**$5238**	**$5.92**	**$18,884**	**$8.88**
Communication with clinical teams—clinical response	$2078	$2.20	$1797	$6.01	$2781	$3.15	$6657	$3.13
Communication with clinical teams—study recruitment	$5570	$5.91	$2793	$9.35	$1969	$2.23	$10,332	$4.86
Context assessment	$579	$0.61	$289	$0.97	$289	$0.33	$1158	$0.54
Update to REDCAP	$228	$0.24	$311	$1.04	$199	$0.23	$737	$0.35
**Clinical champion**	**$817**	**$0.87**	**$1306**	**$4.37**	**$154**	**$0.17**	**$2277**	**$1.07**
Champion training	$344	$0.36	$444	$1.49	$18	$0.02	$807	$0.38
Communication with site lead	$473	$0.50	$861	$2.88	$136	$0.15	$1471	$0.69
**Executive advisory group (EAG)**	**$6906**	**$7.32**	**$10,042**	**$33.60**	**$9649**	**$10.91**	**$26,597**	**$12.51**
EAG meetings	$6799	$7.21	$9143	$30.59	$9439	$10.68	$25,381	$11.94
Meeting with EAG member	$61	$0.06	$567	$1.90	$192	$0.22	$820	$0.39
Set-up of EAG meetings	$45	$0.05	$332	$1.11	$18	$0.02	$395	$0.19
**Facilitation**	**$18,689**	**$19.82**	**$16,865**	**$56.43**	**$6331**	**$7.16**	**$51,962**	**$24.44**
Assisting auditors with auditing	$3040	$3.22	$5929	$19.84	$2316	$2.62	$11,285	$5.31
Auditor inter-reliability	$509	$0.54	$313	$1.05	$248	$0.28	$1070	$0.50
Auditor training	$3155	$3.35	$4590	$15.36	$1637	$1.85	$9382	$4.41
Auditing (in absence of study auditor)	$3184	$3.38	$3429	$11.47	$470	$0.53	$7084	$3.33
Communication with clinical teams—audit enquiry	$287	$0.30	$349	$1.17	$190	$0.21	$826	$0.39
Communication with clinical teams—study information	$7546	$8.00	$1299	$4.35	$916	$1.04	$9761	$4.59
Monitoring- auditing	$968	$1.03	$956	$3.20	$554	$0.63	$2478	$1.17
Printing and materials							$4634	$2.18
Travel							$5442	$2.56
**Total**	**$34,867**	**$37**	**$33,403**	**$112**	**$21,373**	**$24**	**$99,720**	**$47**

^a^These totals include additional costs associated with printing, materials and travel that were not able to be disaggregated at the site level.

**Figure 1:**
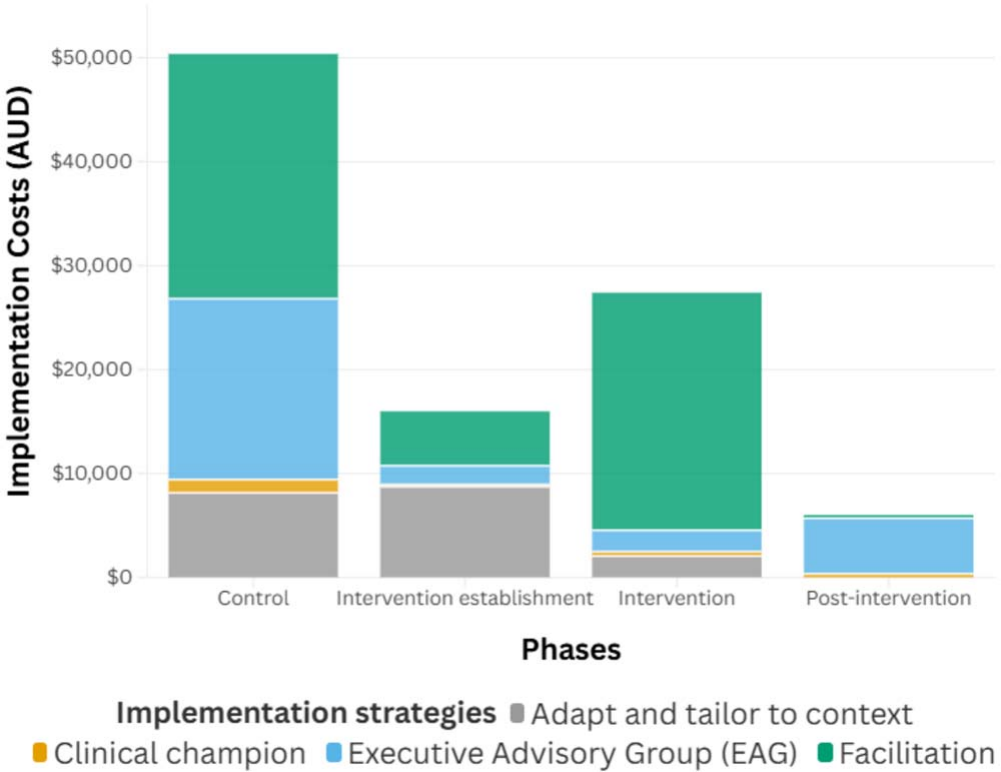
Implementation costs by study phase and implementation strategy

Implementation costs per hospital ranged from $21,373 in Hospital Z to $34,867 in Hospital X (excluding travel and materials costs that were not available at the hospital level). This translated to a cost of $23 per at-risk admission throughout the full duration of the trial. Supplementary Fig. S2 shows implementation costs by hospital and study phase. Hospital-level differences are partly explained by the duration of the intervention exposure phase, which, due to the stepped-wedge study design, was longest in Hospital X and shortest in Hospital Z. Overall, 54% of implementation costs occurred before intervention commencement. A further 19% of costs were incurred during the dedicated intervention establishment phase, with the remaining 28% occurring after the intervention had formally commenced. Figure 1 highlights the cost of different implementation strategies by study phase.

### Hospital admission costs

There were 26 (0.6%) admissions excluded from the admissions costings analysis due to missing cost data. Table 3 outlines the differences in hospital admission costs for the intervention phase, relative to the control phase. After adjusting for calendar time and hospital site cluster, at-risk admissions were relatively more costly during the intervention phase. Conversely, mean readmissions costs were lower. This translated to an overall increase in admission costs of $482 per patient at-risk. However, wide confidence intervals that cross zero indicate that both the direction and magnitude of these estimates are highly uncertain.

**Table 3:** Admission cost differences (AUD) for intervention versus control phases

Admission cost per patient ‘at-risk’	Unadjusted mean difference	Adjusted mean difference	95% CI, adjusted mean difference
At-risk admission	133	524	−2264 to 3312
Readmissions	−1198	−44	−1043 to 955
Total admissions	−1064	482	−1722 to 3651

### Summary of intervention costs and consequences

Table 4 summarises the intervention costs and consequences for every 1000 at-risk patients. When considering the adjusted mean differences, index admission costs were $523,655 higher in the intervention arm (95% CI: –$2.2 million to $3.3 million). These costs were partially offset by $44,193 (95% CI: –$1.0 million to $954,682) in reduced readmissions costs. The overall adjusted mean cost difference, taking account of both intervention and all admission costs, was an additional $558,442 per 1000 patients in the intervention phase. This result was highly uncertain as indicated by the wide 95% confidence interval that crossed zero. The predicted probability of the intervention being cost-increasing was 54%.

**Table 4:** Intervention costs and consequences for every 1000 at-risk admissions

Outcomes	Adjusted mean difference	95% CI, adjusted mean difference
**Cost outcomes (AUD)**		
InterACT intervention costs^	72,219	
Index admission	523,655	−2.2 million to 3.3 million
Readmissions at 12 weeks	−44,193	−1.0 million to 954,682
**Total intervention and admission costs**	**558,442**	**−1.6 million to 3.9 million**
**Clinical and health service outcomes (patient numbers)**		
ICU admission	−12	−75 to 17
Discharged alive	0	−2 to 2
Death in hospital	74	−14 to 138
Medical emergency call	−11	−57 to 38
Hospital readmission at 12 weeks	−56	−91 to −20
**Care review outcomes (patient numbers)** **^a^**		
Clinician led care review discussion		
Hospital X	−30	−68 to 70
Hospital Y	−203	−280 to 124
Hospital Z	25	−23 to 74
Review of care directive measures		
Hospital X	−81	−119 to −43
Hospital Y	−139	−215 to −66
Hospital Z	10	−30 to 51
Specialist palliative care referral		
Hospital X	21	−13 to 56
Hospital Y	−12	−73 to 46
Hospital Z	33	−16 to 82

^ This cost estimate is based on applying the average proportion of at-risk patients being admitted (58%). It would be higher if the proportion at-risk were lower.

^a^Care review outcomes are presented at the hospital subgroup level due to the level of heterogeneity in these results.

Further uncertainty was seen in clinical and health service outcomes, with no clear intervention effect observed for most outcomes. Care review outcomes have been reported at the hospital level due to substantial heterogeneity between sites. The intervention appeared to have a positive impact on reviews of care directive measures at Hospital Z, while the opposite effect was seen in Hospitals X and Y. More detailed findings on clinical and care review outcomes have been published elsewhere [11, 12].

## Discussion

InterACT was a relatively low-cost, light-touch intervention. Our findings suggest it did not impact hospital admissions costs, with wide confidence intervals indicating large variation and uncertainty. This is not surprising given previously reported study findings that showed no statistically significant intervention effect for the trial’s primary endpoint (ICU admissions), or for most of the secondary clinical, health service and care review outcomes [11, 12]. The trial’s process evaluation found that key barriers to effectiveness were: the limited potency of the nudge intervention and its integration into routine clinical practice; clinician beliefs and perceived self-efficacy; and wider contextual factors at the health system level, including the onset of the COVID-19 pandemic [23].

We are not aware of any previous studies assessing the economic value of interventions to reduce non-beneficial treatment at the end of life, although there is evidence that reducing non-beneficial interventions in the ICU is associated with reduced hospital costs [24]. Previous research has found that the nature of incentive structures within Australia’s activity-based healthcare funding system can influence hospital decision makers to value outcomes such as bed days in ways that are inconsistent with the associated financial costs [25]. The cost-consequence approach is therefore well suited here to guide real-world decision-making that accounts for multiple priorities.

The detailed and prospective costing of implementation strategies and activities is a key strength of this study. Implementation strategies can be defined as a method or technique used to enhance the adoption, implementation and sustainability of an intervention [26]. These costs are commonly excluded from economic evaluations [27], which may be due to their lack of visibility, or the intangible nature of these costs which are often absorbed within existing roles or resources. The increasing number of hybrid implementation-effectiveness trials has highlighted the need for stronger health economic methods to understand and quantify the costs associated with implementation strategies. Recent studies have identified the challenges involved in estimating implementation costs in practice and produced recommended guidelines for researchers [28].

The CriSTAL tool was validated for short term death prediction in older emergency department in Australia and Denmark [29]. In both country models CriSTAL had optimal sensitivity (≥ 90%) at low death probabilities of 3%–4% and optimal specificity (> 90%) at higher death probabilities above 20% [30]. However, a key limitation of this study relates to the appropriateness of the trial’s primary outcome, ICU admissions, as a proxy for non-beneficial treatment. This outcome was selected for pragmatic reasons given the size of the trial and the need for routinely collected data items. However, it relies on an inherent assumption that a reduction in ICU admissions represents higher quality of care for patients nearing the end of life. This is untested and may be an over-simplification. The intervention did not improve other secondary outcomes we measured such as clinician-led care review discussions, review of care directive measures and palliative care referrals. Regardless, it remains uncertain whether the study outcomes were sensitive enough to detect more nuanced or disease-specific changes in clinician behaviour, e.g. reductions in initiation or continuation of chemotherapy or radiotherapy in the last month of life. A more detailed analysis of specific treatments may have been better able to detect such impacts.

There are some other limitations of this study to note. First, it was not feasible to collect data on patient-reported outcomes or experience measures, which are important indicators of quality of care and overall value. The consumer perspective is therefore lacking in our analysis. Second, the confidence intervals for many results were wide, indicating uncertainty in our estimates. This was despite the large sample size of the trial which screened over 7000 patients. Third, our analysis of health system costs was limited to the hospital setting, and did not account for costs of follow-up care in the community or primary care settings. Fourth, while implementation costs were recorded prospectively throughout the trial in documents such as field notes and meeting minutes, the choice to apply the Cost-IS instrument in collating and analysing costs was decided on after the trial had ended, due to this instrument only recently becoming available [17]. This presented challenges in categorising some areas of resource use, with simplifying assumptions needing to be made. For example, study investigator meeting times were excluded, as it was not possible to determine whether discussion related to implementation or the research project more broadly. In addition, as travel related costs were not documented at the hospital level, these have been excluded from hospital-specific costings and only reported in aggregate. We recommend that future trials looking to cost implementation strategies plan to embed a tool such as Cost-IS into prospective data collection activities to optimise its utility.

The substantial costs associated with hospital admissions for people nearing the end of life highlight the need for ongoing efforts to develop and test new interventions to minimise low value care. Our findings suggest that the simple nudge-style InterACT intervention, despite its relatively low cost, was not sufficient to impact health service resource use in the complex end-of-life setting. Future studies should investigate the economic value of more active, multi-component interventions.

## Supplementary Material

aa-25-0895-File002_afaf280

## Data Availability

The full data can be requested using the QCIF dataverse: https://doi.org/10.60540/PT6IPY.
