# Supplementary material for: A cost-consequence analysis of a nudge intervention to improve hospital care of older people at the end of life: results from a stepped-wedge cluster randomised trial
Source: Age Ageing. 2025 Oct 6;54(10):afaf280. doi: 10.1093/ageing/afaf280 (PMC12499750; doi:10.1093/ageing/afaf280)
Supplement: aa-25-0895-File002_afaf280 [file aa-25-0895-file002_afaf280.docx]

**A cost-consequence analysis of a nudge intervention to improve hospital care of older people at the end of life: results from a stepped-wedge cluster randomised trial**

**SUPPLEMENTARY MATERIALS**

**Contents:**

| **Document** | **Page number** |
| --- | --- |
| File S1: The InterACT Intervention: additonal information on the screening process | 2 |
| Figure S1: Stepped-wedge design showing the control and intervention periods in the three hospitals | 3 |
| Figure S2: Implementation costs by hospital and study phase | 4 |
| Table S1: Descriptive table of included patient admissions for the control and intervention phases. Q1 to Q3 is the first to third quartile | 5 |

**File S1: The InterACT Intervention: additonal information on the screening process**

Both SPICT and CriSTAL risk screening tools were applied concurrently to the records and eligible patients were those screening ‘at risk of death’ by either tool. The SPICT predicting risk of death within one year was familiar to the majority of clinicians, general medicine, involved in the study as it was developed in the UK and widely used by others. However, it requires clinical judgment by expert clinicians and as auditors did not have access to patients, the use of the SPICT was limited to the general indicators. By contrast, the CriSTAL tool was developed by Australian co-investigators, also validated nationally and internationally and its scoring is based on objective parameters from the existing medical record, not requiring further patient examination. Some of the risk factors measured with in the CriSTAL tool overlapped with the clinical indicators and life-limiting conditions from the second section of the SPICT tool, therefore we believed using both tools provided more comprehensive screening and additional assessment of short-term risk.

Patients were classified as at-risk with a CriSTAL score of 6 or greater and/ or a SPICT score of 2 or greater. The flag was designed as an alert to clinicians, who were familiar with the screening tools and aware of their accuracy. There was a two-fold notification system. The first notification was a real-time alert, either attached to the patient electronic record or medical handover report. The second notification was an audit report email sent to clinicians caring for at-risk patients at the end of each screening day. This email contained the items measured in each screening tool that were present for the patient and their current health state, i.e. clinical frailty score of 5 or more. The idea of the nudge was to prompt the clinicians to consider changes in care. It was not prescriptive and did not interfere with routine clinical practice. However, clinicians were aware of the study objectives, and they understood the rationale for reducing non-beneficial treatments.

Over 7,000 admissions were screened during the trial, with 4,268 patients identified as being ‘at-risk’ of receiving non-beneficial treatment based on their likelihood of being in, or approaching, their end of life.

Figure S1: Stepped-wedge design showing the control and intervention periods in the three hospitals


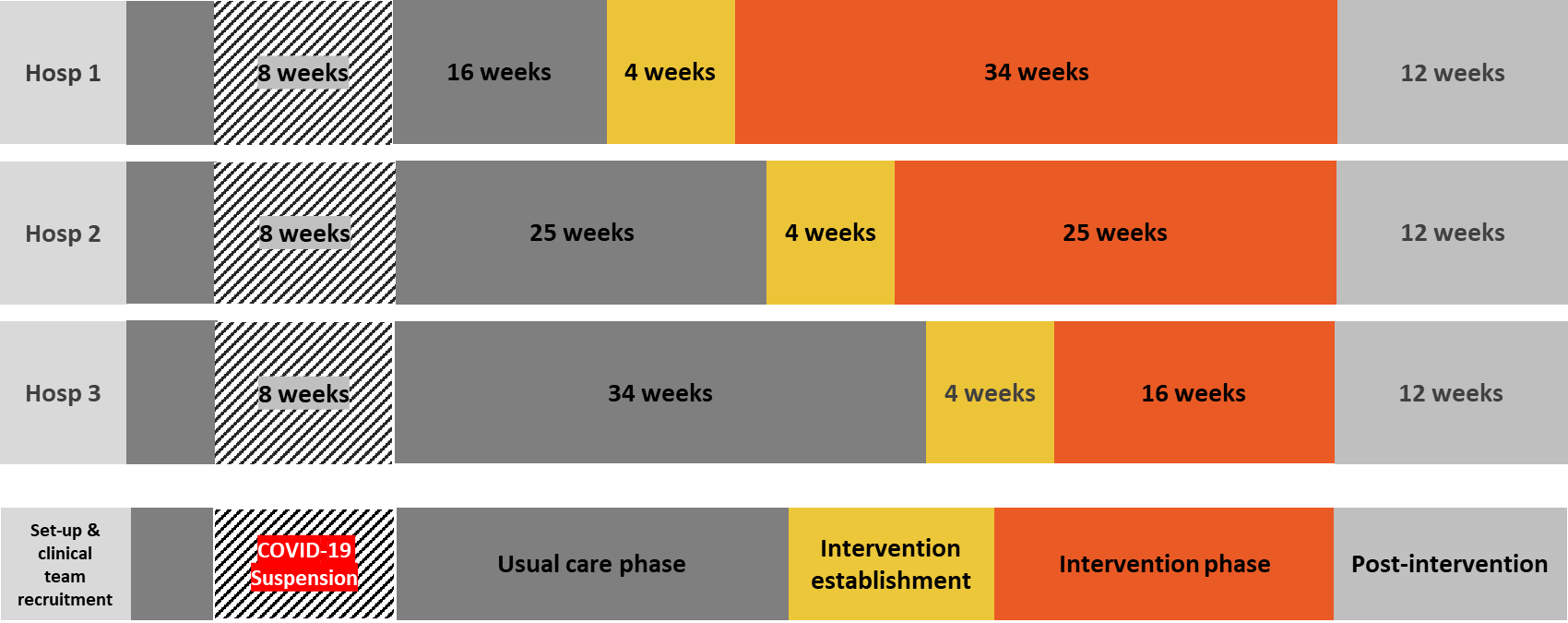


Figure S2: Implementation costs by hospital and study phase*


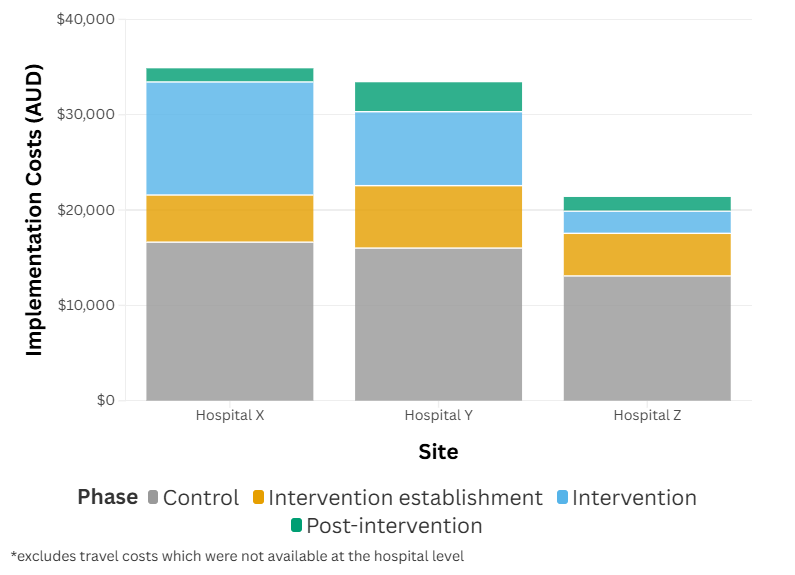


Table S1: Descriptive table of included patient admissions for the control and intervention phases. Q1 to Q3 is the first to third quartile.

|  | Control | Intervention |
| --- | --- | --- |
| Weeks | 75 | 75 |
| Number at risk | 2,142 | 2,126 |
| Admission dates, median [Q1 to Q3] | Aug 2020  [Jul 2020 to Oct 2020] | Feb 2021  [Dec 2020 to Apr 2021] |
| Female, n (%) | 1,119 (52%) | 1,186 (56%) |
| Age, median [Q1 to Q3] | 84 [79 to 88] | 84 [79 to 89] |
| CriSTAL score, median [Q1 to Q3] | 5 [4 to 6] | 5 [4 to 6] |
| SPICT score, median [Q1 to Q3] | 2 [2 to 3] | 3 [2 to 3] |
| Length of stay, median [Q1 to Q3] | 5.8 [3.2 to 11.1] | 6.0 [3.3 to 12.4] |
